# 2-(2-Ammonio­ethyl)pyridinium hexa­chloridorhenate(IV)

**DOI:** 10.1107/S1600536808044097

**Published:** 2009-02-04

**Authors:** Andrzej Kochel

**Affiliations:** a Faculty of Chemistry, University of Wrocław, 14 Joliot-Curie Street, 50-383 Wrocław, Poland

## Abstract

In the title anti­ferromagnetic material, (C_7_H_12_N_2_)[ReCl_6_], the Néel temperature is observed at 5 K. The salt is stabilized by an extensive network of N—H⋯Cl and C—H⋯Cl hydrogen bonds, where hydrogen-bonded anion chains and characteristic cation–anion motifs are present. Similar systems play an important role in crystal engineering as hydrogen bonds that can transmit magnetic inter­actions.

## Related literature

For related literature, see: Kepert *et al.* (1997 ▶); Mrozinski *et al.* (2002 ▶); Sawusch & Schilde (1999 ▶); Kochel (2004 ▶); Koenig (1966 ▶).
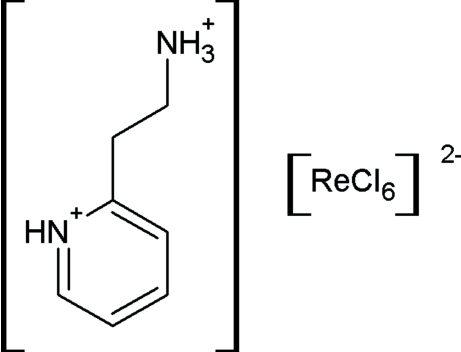


## Experimental

### 

#### Crystal data

(C_7_H_12_N_2_)[ReCl_6_]
*M* *_r_* = 523.10
Triclinic, 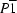

*a* = 7.371 (2) Å
*b* = 14.204 (3) Å
*c* = 15.159 (3) Å
α = 66.87 (2)°
β = 84.74 (2)°
γ = 75.61 (2)°
*V* = 1413.7 (6) Å^3^
*Z* = 4
Mo *K*α radiation
μ = 9.70 mm^−1^
*T* = 100 (2) K
0.14 × 0.10 × 0.06 mm

#### Data collection

Oxford Diffraction KM-4-CCD diffractometer
Absorption correction: analytical (*CrysAlis RED*; Oxford Diffraction, 2006 ▶) *T*_min_ = 0.367, *T*_max_ = 0.559
21570 measured reflections
10147 independent reflections
8098 reflections with *I* > 2σ(*I*)
*R*_int_ = 0.023

#### Refinement

*R*[*F* ^2^ > 2σ(*F* ^2^)] = 0.024
*wR*(*F* ^2^) = 0.053
*S* = 0.98
10147 reflections
313 parameters
H atoms treated by a mixture of independent and constrained refinement
Δρ_max_ = 2.07 e Å^−3^
Δρ_min_ = −1.31 e Å^−3^

### 

Data collection: *CrysAlis CCD* (Oxford Diffraction, 2006 ▶); cell refinement: *CrysAlis RED* (Oxford Diffraction, 2006 ▶); data reduction: *CrysAlis RED*; program(s) used to solve structure: *SHELXS97* (Sheldrick, 2008 ▶); program(s) used to refine structure: *SHELXL97* (Sheldrick, 2008 ▶); molecular graphics: *ORTEP-3* (Farrugia, 1997 ▶); software used to prepare material for publication: *SHELXL97*.

## Supplementary Material

Crystal structure: contains datablocks I, global. DOI: 10.1107/S1600536808044097/bv2112sup1.cif

Structure factors: contains datablocks I. DOI: 10.1107/S1600536808044097/bv2112Isup2.hkl

Additional supplementary materials:  crystallographic information; 3D view; checkCIF report

## Figures and Tables

**Table 1:** Hydrogen-bond geometry (Å, °)

*D*—H⋯*A*	*D*—H	H⋯*A*	*D*⋯*A*	*D*—H⋯*A*
N1—H1⋯Cl3^i^	0.86	2.76	3.280 (3)	120
N1—H1⋯Cl12^ii^	0.86	2.56	3.208 (3)	133
N2—H2*N*⋯Cl1^iii^	0.93 (4)	2.47 (4)	3.313 (3)	152 (3)
N3—H3*A*⋯Cl7^iv^	0.86	2.71	3.431 (3)	142
N3—H3*A*⋯Cl12^iv^	0.86	2.65	3.326 (3)	136
N2—H3*N*⋯Cl1^i^	0.83 (5)	2.50 (5)	3.328 (3)	174 (4)
N4—H6*N*⋯Cl2	0.93 (6)	2.43 (5)	3.291 (4)	155 (4)
N4—H9*N*⋯Cl7	1.04 (6)	2.70 (5)	3.199 (3)	110 (3)
N4—H9*N*⋯Cl8	1.04 (6)	2.52 (6)	3.541 (3)	166 (4)
N4—H10*N*⋯Cl11^iii^	0.87 (5)	2.45 (4)	3.240 (4)	152 (4)
C4—H4⋯Cl11	0.93	2.83	3.621 (3)	144
C7—H7*B*⋯Cl1^v^	0.97	2.80	3.711 (3)	156
C22—H22⋯Cl2^vi^	0.93	2.69	3.602 (4)	166
C26—H26*B*⋯Cl8	0.97	2.82	3.589 (3)	137
C27—H27*B*⋯Cl5	0.97	2.81	3.610 (3)	140

Symmetry codes: (i) 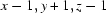; (ii) 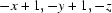; (iii) 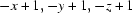; (iv) 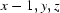; (v) 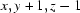; (vi) 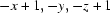.

**Fig. 1.**
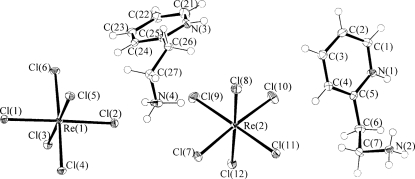
Molecular structure of (1). Thermal ellipsoids are drawn at 50% probability level.

**Fig. 2.**
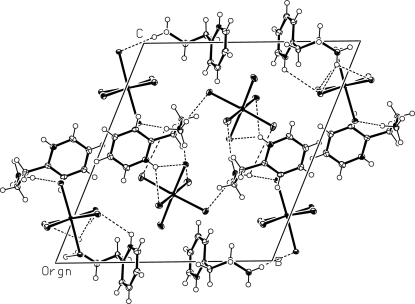
The crystal structure packing scheme viewed along [100] showing the hydrogen bonds system.

**Fig. 3.**
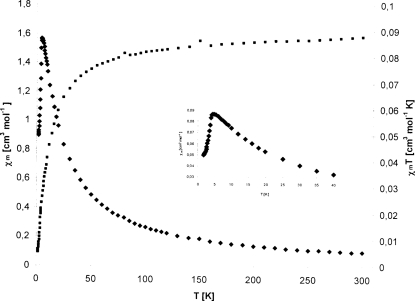
The χm and χmT temperature dependence (χm - the molar magnetic susceptibility) for compund (1).

## supplementary crystallographic information

### Comment

Rhenium(IV) salts with ammonium cations are known and have been described previously (Sawusch *et al.*, 1999, Mrozinski *et al.*, 2002, Kochel 2004). Some of the hexachlororhenates(IV) have interesting properties, *e.g.* as semiconductors (Kepert *et al.*, 1997). The title salt comprises of 2-(2-aminoethyl)pyridinium cations and ReCl_6_^2-^ anions. Figure 1 illustrates the two independent formula units of compound (1). The anion bond lengths are comparable to those for other anions of this type (see table in supplementary material). The crystal structure is stabilized by an extensive network of N—H···Cl and C—H···Cl hydrogen bonds (Fig. 2). Almost all amino H atoms bonded to the organic cation participate in hydrogen bonds as donors. Some of the hydrogen bonds *e.g.* N3—H3···Cl12, N3—H3···Cl1 are bifurcated. The hydrogen bonding parameters are included in Table 1. The use of small low-symmetry ammonium cations enable these ions to occupy general positions, so that the Re···Re distances are much smaller. In the crystal packing, two types of arrangement of molecules may be distinguished: in the [100] direction a layered arrangement [alternating anions and cations] is observed, while in the [010] direction, and the whole is stabilized by a network of hydrogen bonds. The shortest Re1···Re1_i_ distance is 7.371 (2)Å [symmetry code: (i) *x* - 1,*y*,*z*]. The magnetic susceptibility of (1) is measured in the temperature range from 2 to 300 K under the applied magnetic field of 0.5 T (Figure 3). The χ_m_T values decrease slowly upon cooling. The effective magnetic moment of 3.55 B. *M*. at 300 K is reduced in comparison to the spin-only value (3.87 uB), and in 2 K is 0.84 B. *M*. Generally, the complex shows linear χ_m_*versus* T behaviour in the 300–50 K range with C = 1.70 cm^3^ mol^-1^ K, Q = -14.5 K. The susceptibility curves exhibit the maximum at 5 K, indicating directly the presence of antiferromagnetic interactions.

The χ_m_T at 300 K is 1.58 cm^3^ K_mol_^-1^ and this value is expected for an isolated Re(IV) ion. As the temperature is lowered, χ_m_T decreases, and the value is 0.896 cm^3^ K_mol_^-1^ at 2 K. This behaviour indicates antiferromagnetic interactions between the Re(IV) ions. The occurrence of antiferromagentic interactions could be related to aspects of the structure. The smallest Re—Re distance is 7.371 (2) Å, and a substantial value, however, there are numerous hydrogen bonds in the crystal structure, forming a three-dimensional framework. The hydrogen bonds stabilize the crystal structure layered arrangement. Probably the hydrogen bonding system enhances the magnetic exchange interactions. Future perspectives concerning this work will involve further studies on hexachlororhenates(IV) as potential materials used as semiconductors.

### Experimental

(NH_4_)_2_ReCl_6_ (0.15 g) was dissolved in water (50 ml) with concentrated HCl (2 ml) and the mixture was heated under reflux at 340 K. After 30 min, 2-(2-aminoethyl)pyridine (0.25 g) was added. The mixture was heated for further 5 h. After cooling, the yellow precipitate was filtered off and washed with ethanol. Crystals for X-ray study were obtained by slow evaporation of an aqueous solution of the yellow precipitate with addition of 1 ml HCl solution. The crystals are in the form of plates. For data collection a small plate was used, cut from a larger one.

Elemental analysis of C, H, Cl, and N for C7 H12 Cl6 N2 Re1: calcd. for C, 16.07; H, 2.31; Cl, 40.66; found: C, 15.03; H, 2.01; Cl, 39.12;

IR spectra were collected for samples prepared as KBr pellets on a BRUKER spectrometer.

(1) 3456 (*s*), 3010 (*s*), 2574 (*m*), 2015 (*m*), 1610 (*m*), 1545 (*s*), 1431 (*s*), 1403 (*m*), 1321 (*s*), 1120 (*s*), 935 (*s*), 715 (*versus*), 645 (*versus*), 554 (*versus*), 435 (*versus*), 303 (*m*), 295 (*versus*), 211 (*versus*), 174 (*versus*), 160 (*versus*).

The magnetic measurements of polycrystalline samples were carried out over the temperature range of 2–300 K using a Quantum design SQUID-Based Magnetometer MPMSXL5. The SQUID magnetometer was calibrated with a palladium rod sample, for which the gram susceptibility was assumed as 5.30 x 10^–6^ cm^3^g^-1^ at 293 K (National Bureau of Standards, USA). The susceptibility measurements were made in the field of 0.5 T. Corrections were done for the diamagnetic response of the sample rod and of the sample using Pascal's constants (Koenig, 1966).

### Refinement

The structure (1) was solved by direct methods using *SHELXS97* software (Sheldrick, 2008) and refined using *SHELXL97* (Sheldrick, 2008). In case of (1) *DFIX* restraints were used for all C—H bond lengths (0.93–0.97 Å with the allowed deviation of 0.002 Å. All H atoms were refined with *U*_eq_ set at 1.2 *U*_eq_ (parent atom). H atoms associated with N atoms were located on difference maps and then freely refined. In the final difference maps the following highest peaks were found: for (1) the maximum of -1.30 and 2.07 e/Å^3^ at 0.68 and 0.78 Å from the Re(2) atom

### Crystal data

**Table:**

(C_7_H_12_N_2_)[ReCl_6_]	*Z* = 4
*M**_r_* = 523.10	*F*(000) = 980
Triclinic, *P*1	*D*_x_ = 2.458 Mg m^−^^3^
Hall symbol: -P 1	Mo *K*α radiation, λ = 0.71073 Å
*a* = 7.371 (2) Å	Cell parameters from 7350 reflections
*b* = 14.204 (3) Å	θ = 2.7–36.9°
*c* = 15.159 (3) Å	µ = 9.70 mm^−^^1^
α = 66.87 (2)°	*T* = 100 K
β = 84.74 (2)°	Needle, yellow
γ = 75.61 (2)°	0.14 × 0.10 × 0.06 mm
*V* = 1413.7 (6) Å^3^	

### Data collection

**Table:**

Oxford Diffraction KM-4-CCD diffractometer	10147 independent reflections
Radiation source: fine-focus sealed tube	8098 reflections with *I* > 2σ(*I*)
graphite	*R*_int_ = 0.023
/w scans	θ_max_ = 36.9°, θ_min_ = 2.7°
Absorption correction: analytical (*CrysAlis RED*; Oxford Diffraction, 2006)	*h* = −12→10
*T*_min_ = 0.367, *T*_max_ = 0.559	*k* = −23→18
21570 measured reflections	*l* = −25→25

### Refinement

**Table:**

Refinement on *F*^2^	Primary atom site location: structure-invariant direct methods
Least-squares matrix: full	Secondary atom site location: difference Fourier map
*R*[*F*^2^ > 2σ(*F*^2^)] = 0.024	Hydrogen site location: inferred from neighbouring sites
*wR*(*F*^2^) = 0.053	H atoms treated by a mixture of independent and constrained refinement
*S* = 0.98	*w* = 1/[σ^2^(*F*_o_^2^) + (0.0289*P*)^2^] where *P* = (*F*_o_^2^ + 2*F*_c_^2^)/3
10147 reflections	(Δ/σ)_max_ = 0.001
313 parameters	Δρ_max_ = 2.07 e Å^−^^3^
0 restraints	Δρ_min_ = −1.31 e Å^−^^3^

### Special details

**Table:**

Geometry. All e.s.d.'s (except the e.s.d. in the dihedral angle between two l.s. planes) are estimated using the full covariance matrix. The cell e.s.d.'s are taken into account individually in the estimation of e.s.d.'s in distances, angles and torsion angles; correlations between e.s.d.'s in cell parameters are only used when they are defined by crystal symmetry. An approximate (isotropic) treatment of cell e.s.d.'s is used for estimating e.s.d.'s involving l.s. planes.
Refinement. Refinement of *F*^2^ against ALL reflections. The weighted *R*-factor *wR* and goodness of fit *S* are based on *F*^2^, conventional *R*-factors *R* are based on *F*, with *F* set to zero for negative *F*^2^. The threshold expression of *F*^2^ > σ(*F*^2^) is used only for calculating *R*-factors(gt) *etc*. and is not relevant to the choice of reflections for refinement. *R*-factors based on *F*^2^ are statistically about twice as large as those based on *F*, and *R*- factors based on ALL data will be even larger.

### Fractional atomic coordinates and isotropic or equivalent isotropic displacement parameters (Å^2^)

**Table:**

	*x*	*y*	*z*	*U*_iso_*/*U*_eq_	
N1	0.0792 (4)	0.6798 (2)	−0.06898 (17)	0.0146 (5)	
H1	0.1179	0.7004	−0.1273	0.018*	
N2	0.2688 (4)	0.9123 (2)	−0.05025 (19)	0.0160 (5)	
C1	−0.0762 (4)	0.6422 (3)	−0.0503 (2)	0.0182 (6)	
H1A	−0.1404	0.6400	−0.0992	0.022*	
C2	−0.1379 (4)	0.6072 (3)	0.0424 (2)	0.0189 (7)	
H2	−0.2442	0.5798	0.0575	0.023*	
C3	−0.0401 (4)	0.6128 (3)	0.1141 (2)	0.0177 (6)	
H3	−0.0806	0.5888	0.1773	0.021*	
C4	0.1171 (4)	0.6543 (3)	0.0911 (2)	0.0160 (6)	
H4	0.1809	0.6597	0.1384	0.019*	
C5	0.1791 (4)	0.6877 (2)	−0.0029 (2)	0.0126 (5)	
C6	0.3564 (4)	0.7241 (3)	−0.0333 (2)	0.0161 (6)	
H6A	0.3593	0.7542	−0.1028	0.019*	
H6B	0.4610	0.6633	−0.0116	0.019*	
C7	0.3845 (4)	0.8048 (3)	0.0037 (2)	0.0160 (6)	
H7A	0.3516	0.7832	0.0711	0.019*	
H7B	0.5157	0.8070	−0.0017	0.019*	
N3	0.0641 (4)	0.2257 (2)	0.46864 (18)	0.0164 (5)	
H3A	−0.0082	0.2847	0.4348	0.020*	
N4	0.3743 (4)	0.3282 (3)	0.6043 (2)	0.0194 (6)	
C21	0.1078 (5)	0.1501 (3)	0.4337 (2)	0.0213 (7)	
H21	0.0610	0.1621	0.3744	0.026*	
C22	0.2226 (5)	0.0542 (3)	0.4860 (2)	0.0188 (6)	
H22	0.2526	0.0003	0.4631	0.023*	
C23	0.2926 (4)	0.0396 (3)	0.5741 (2)	0.0164 (6)	
H23	0.3720	−0.0240	0.6101	0.020*	
C24	0.2437 (4)	0.1201 (3)	0.6074 (2)	0.0145 (6)	
H24	0.2901	0.1102	0.6662	0.017*	
C25	0.1265 (4)	0.2152 (2)	0.55434 (19)	0.0136 (6)	
C26	0.0569 (4)	0.3072 (3)	0.5838 (2)	0.0144 (6)	
H26A	−0.0620	0.3012	0.6162	0.017*	
H26B	0.0338	0.3712	0.5264	0.017*	
C27	0.1872 (4)	0.3179 (3)	0.6489 (2)	0.0155 (6)	
H27A	0.1309	0.3793	0.6637	0.019*	
H27B	0.2037	0.2566	0.7086	0.019*	
Re1	0.774116 (15)	0.011796 (9)	0.795978 (7)	0.00979 (3)	
Cl1	0.82082 (10)	−0.09836 (6)	0.96174 (4)	0.01273 (13)	
Cl2	0.72966 (10)	0.12498 (6)	0.63386 (5)	0.01403 (13)	
Cl3	1.05734 (10)	−0.08752 (6)	0.75988 (5)	0.01415 (13)	
Cl4	0.93941 (10)	0.12262 (6)	0.82086 (5)	0.01445 (14)	
Cl5	0.48903 (10)	0.10735 (6)	0.83435 (5)	0.01483 (14)	
Cl6	0.61222 (10)	−0.10277 (6)	0.77706 (5)	0.01580 (14)	
Re2	0.540061 (16)	0.419170 (10)	0.306599 (7)	0.01145 (3)	
Cl7	0.66713 (10)	0.42137 (6)	0.44425 (5)	0.01578 (14)	
Cl8	0.23337 (11)	0.46658 (7)	0.36098 (5)	0.02068 (16)	
Cl9	0.54298 (12)	0.24161 (7)	0.38861 (6)	0.02359 (17)	
Cl10	0.43132 (12)	0.42195 (7)	0.16505 (6)	0.02313 (17)	
Cl11	0.53303 (10)	0.60222 (6)	0.22842 (5)	0.01651 (14)	
Cl12	0.85405 (10)	0.37428 (7)	0.25762 (5)	0.01766 (15)	
H2N	0.290 (5)	0.961 (3)	−0.028 (3)	0.019 (9)*	
H3N	0.155 (6)	0.915 (3)	−0.050 (3)	0.029 (11)*	
H4N	0.298 (5)	0.933 (3)	−0.107 (3)	0.024 (10)*	
H6N	0.444 (6)	0.262 (4)	0.610 (3)	0.035 (12)*	
H9N	0.346 (6)	0.378 (4)	0.533 (4)	0.047 (14)*	
H10N	0.440 (6)	0.341 (3)	0.641 (3)	0.024 (10)*	

### Atomic displacement parameters (Å^2^)

**Table:**

	*U*^11^	*U*^22^	*U*^33^	*U*^12^	*U*^13^	*U*^23^
N1	0.0165 (12)	0.0168 (13)	0.0139 (11)	−0.0070 (11)	0.0019 (9)	−0.0079 (10)
N2	0.0175 (14)	0.0154 (14)	0.0168 (12)	−0.0064 (12)	0.0007 (10)	−0.0066 (11)
C1	0.0161 (15)	0.0213 (17)	0.0184 (13)	−0.0066 (14)	−0.0016 (11)	−0.0071 (13)
C2	0.0172 (15)	0.0171 (16)	0.0214 (14)	−0.0071 (14)	0.0024 (11)	−0.0048 (13)
C3	0.0172 (15)	0.0170 (16)	0.0178 (13)	−0.0030 (13)	0.0001 (11)	−0.0059 (12)
C4	0.0167 (14)	0.0161 (15)	0.0146 (12)	−0.0040 (13)	−0.0007 (10)	−0.0048 (12)
C5	0.0132 (13)	0.0104 (14)	0.0135 (12)	−0.0015 (12)	0.0006 (10)	−0.0046 (11)
C6	0.0129 (14)	0.0154 (15)	0.0217 (14)	−0.0023 (13)	0.0009 (11)	−0.0096 (13)
C7	0.0143 (14)	0.0149 (15)	0.0194 (13)	−0.0019 (13)	−0.0042 (10)	−0.0071 (12)
N3	0.0154 (12)	0.0187 (14)	0.0151 (11)	−0.0031 (11)	−0.0031 (9)	−0.0063 (11)
N4	0.0157 (13)	0.0189 (15)	0.0286 (14)	−0.0056 (12)	0.0005 (11)	−0.0135 (13)
C21	0.0271 (17)	0.0229 (18)	0.0180 (14)	−0.0059 (15)	−0.0026 (12)	−0.0115 (14)
C22	0.0197 (15)	0.0181 (16)	0.0218 (14)	−0.0040 (14)	0.0000 (12)	−0.0112 (13)
C23	0.0166 (14)	0.0141 (15)	0.0181 (13)	−0.0042 (13)	−0.0003 (11)	−0.0053 (12)
C24	0.0141 (14)	0.0172 (16)	0.0115 (11)	−0.0075 (13)	0.0016 (10)	−0.0027 (11)
C25	0.0126 (14)	0.0158 (15)	0.0136 (12)	−0.0047 (13)	0.0008 (10)	−0.0062 (12)
C26	0.0126 (13)	0.0145 (15)	0.0171 (12)	−0.0038 (12)	−0.0008 (10)	−0.0064 (12)
C27	0.0157 (14)	0.0167 (16)	0.0156 (12)	−0.0028 (13)	0.0005 (10)	−0.0083 (12)
Re1	0.01005 (5)	0.01098 (6)	0.00933 (4)	−0.00349 (5)	0.00008 (3)	−0.00430 (4)
Cl1	0.0146 (3)	0.0142 (3)	0.0096 (3)	−0.0050 (3)	0.0005 (2)	−0.0040 (2)
Cl2	0.0158 (3)	0.0139 (3)	0.0114 (3)	−0.0034 (3)	−0.0010 (2)	−0.0037 (3)
Cl3	0.0135 (3)	0.0146 (3)	0.0139 (3)	−0.0017 (3)	0.0017 (2)	−0.0063 (3)
Cl4	0.0148 (3)	0.0152 (3)	0.0159 (3)	−0.0069 (3)	−0.0001 (2)	−0.0065 (3)
Cl5	0.0118 (3)	0.0171 (4)	0.0168 (3)	−0.0028 (3)	0.0013 (2)	−0.0084 (3)
Cl6	0.0183 (3)	0.0174 (4)	0.0159 (3)	−0.0087 (3)	−0.0002 (2)	−0.0079 (3)
Re2	0.01206 (6)	0.01075 (6)	0.01218 (5)	−0.00259 (5)	−0.00074 (4)	−0.00493 (4)
Cl7	0.0174 (3)	0.0174 (4)	0.0127 (3)	−0.0018 (3)	−0.0019 (2)	−0.0069 (3)
Cl8	0.0133 (3)	0.0242 (4)	0.0225 (3)	−0.0029 (3)	0.0022 (3)	−0.0081 (3)
Cl9	0.0262 (4)	0.0128 (4)	0.0297 (4)	−0.0066 (3)	−0.0014 (3)	−0.0044 (3)
Cl10	0.0263 (4)	0.0244 (4)	0.0228 (4)	0.0010 (4)	−0.0093 (3)	−0.0155 (3)
Cl11	0.0193 (3)	0.0126 (3)	0.0170 (3)	−0.0048 (3)	−0.0037 (2)	−0.0034 (3)
Cl12	0.0151 (3)	0.0214 (4)	0.0140 (3)	−0.0007 (3)	0.0017 (2)	−0.0066 (3)

### Geometric parameters (Å, °)

**Table:**

N1—C1	1.342 (4)	N4—H10N	0.87 (4)
N1—C5	1.349 (4)	C21—C22	1.379 (5)
N1—H1	0.8600	C21—H21	0.9300
N2—C7	1.492 (4)	C22—C23	1.396 (4)
N2—H2N	0.94 (4)	C22—H22	0.9300
N2—H3N	0.83 (4)	C23—C24	1.381 (5)
N2—H4N	0.82 (4)	C23—H23	0.9300
C1—C2	1.370 (4)	C24—C25	1.383 (4)
C1—H1A	0.9300	C24—H24	0.9300
C2—C3	1.396 (4)	C25—C26	1.501 (4)
C2—H2	0.9300	C26—C27	1.511 (4)
C3—C4	1.383 (4)	C26—H26A	0.9700
C3—H3	0.9300	C26—H26B	0.9700
C4—C5	1.386 (4)	C27—H27A	0.9700
C4—H4	0.9300	C27—H27B	0.9700
C5—C6	1.492 (4)	Re1—Cl2	2.3480 (9)
C6—C7	1.522 (4)	Re1—Cl6	2.3556 (8)
C6—H6A	0.9700	Re1—Cl5	2.3611 (10)
C6—H6B	0.9700	Re1—Cl3	2.3624 (10)
C7—H7A	0.9700	Re1—Cl4	2.3682 (8)
C7—H7B	0.9700	Re1—Cl1	2.3839 (9)
N3—C21	1.333 (4)	Re2—Cl9	2.3284 (10)
N3—C25	1.359 (4)	Re2—Cl10	2.3412 (9)
N3—H3A	0.8600	Re2—Cl8	2.3624 (10)
N4—C27	1.495 (4)	Re2—Cl12	2.3744 (10)
N4—H6N	0.92 (5)	Re2—Cl7	2.3783 (8)
N4—H9N	1.04 (5)	Re2—Cl11	2.3856 (10)
			
C1—N1—C5	124.7 (2)	C24—C23—C22	119.7 (3)
C1—N1—H1	117.6	C24—C23—H23	120.1
C5—N1—H1	117.6	C22—C23—H23	120.1
C7—N2—H2N	111 (2)	C23—C24—C25	120.7 (3)
C7—N2—H3N	114 (3)	C23—C24—H24	119.6
H2N—N2—H3N	110 (4)	C25—C24—H24	119.6
C7—N2—H4N	111 (3)	N3—C25—C24	117.1 (3)
H2N—N2—H4N	106 (4)	N3—C25—C26	116.7 (3)
H3N—N2—H4N	105 (4)	C24—C25—C26	126.1 (3)
N1—C1—C2	118.5 (3)	C25—C26—C27	115.2 (3)
N1—C1—H1A	120.8	C25—C26—H26A	108.5
C2—C1—H1A	120.8	C27—C26—H26A	108.5
C1—C2—C3	119.5 (3)	C25—C26—H26B	108.5
C1—C2—H2	120.3	C27—C26—H26B	108.5
C3—C2—H2	120.3	H26A—C26—H26B	107.5
C4—C3—C2	120.0 (3)	N4—C27—C26	112.1 (2)
C4—C3—H3	120.0	N4—C27—H27A	109.2
C2—C3—H3	120.0	C26—C27—H27A	109.2
C3—C4—C5	119.7 (3)	N4—C27—H27B	109.2
C3—C4—H4	120.2	C26—C27—H27B	109.2
C5—C4—H4	120.2	H27A—C27—H27B	107.9
N1—C5—C4	117.7 (3)	Cl2—Re1—Cl6	92.04 (3)
N1—C5—C6	118.6 (2)	Cl2—Re1—Cl5	89.96 (4)
C4—C5—C6	123.6 (3)	Cl6—Re1—Cl5	89.52 (3)
C5—C6—C7	115.3 (3)	Cl2—Re1—Cl3	90.99 (4)
C5—C6—H6A	108.4	Cl6—Re1—Cl3	89.50 (3)
C7—C6—H6A	108.4	Cl5—Re1—Cl3	178.66 (2)
C5—C6—H6B	108.4	Cl2—Re1—Cl4	90.04 (3)
C7—C6—H6B	108.4	Cl6—Re1—Cl4	177.91 (2)
H6A—C6—H6B	107.5	Cl5—Re1—Cl4	90.64 (3)
N2—C7—C6	112.2 (2)	Cl3—Re1—Cl4	90.31 (3)
N2—C7—H7A	109.2	Cl2—Re1—Cl1	178.08 (3)
C6—C7—H7A	109.2	Cl6—Re1—Cl1	89.74 (3)
N2—C7—H7B	109.2	Cl5—Re1—Cl1	89.34 (4)
C6—C7—H7B	109.2	Cl3—Re1—Cl1	89.74 (4)
H7A—C7—H7B	107.9	Cl4—Re1—Cl1	88.17 (3)
C21—N3—C25	124.1 (3)	Cl9—Re2—Cl10	92.44 (4)
C21—N3—H3A	118.0	Cl9—Re2—Cl8	90.47 (4)
C25—N3—H3A	118.0	Cl10—Re2—Cl8	92.67 (4)
C27—N4—H6N	109 (3)	Cl9—Re2—Cl12	90.34 (4)
C27—N4—H9N	105 (2)	Cl10—Re2—Cl12	90.12 (3)
H6N—N4—H9N	111 (4)	Cl8—Re2—Cl12	177.06 (3)
C27—N4—H10N	109 (3)	Cl9—Re2—Cl7	89.92 (4)
H6N—N4—H10N	100 (4)	Cl10—Re2—Cl7	176.13 (3)
H9N—N4—H10N	122 (4)	Cl8—Re2—Cl7	90.37 (3)
N3—C21—C22	119.7 (3)	Cl12—Re2—Cl7	86.80 (3)
N3—C21—H21	120.1	Cl9—Re2—Cl11	177.67 (3)
C22—C21—H21	120.1	Cl10—Re2—Cl11	89.43 (4)
C21—C22—C23	118.6 (3)	Cl8—Re2—Cl11	88.05 (4)
C21—C22—H22	120.7	Cl12—Re2—Cl11	91.05 (4)
C23—C22—H22	120.7	Cl7—Re2—Cl11	88.28 (3)

### Hydrogen-bond geometry (Å, °)

**Table:**

*D*—H···*A*	*D*—H	H···*A*	*D*···*A*	*D*—H···*A*
N1—H1···Cl3^i^	0.86	2.76	3.280 (3)	120
N1—H1···Cl12^ii^	0.86	2.56	3.208 (3)	133
N2—H2N···Cl1^iii^	0.93 (4)	2.47 (4)	3.313 (3)	152 (3)
N3—H3A···Cl7^iv^	0.86	2.71	3.431 (3)	142
N3—H3A···Cl12^iv^	0.86	2.65	3.326 (3)	136
N2—H3N···Cl1^i^	0.83 (5)	2.50 (5)	3.328 (3)	174 (4)
N4—H6N···Cl2	0.93 (6)	2.43 (5)	3.291 (4)	155 (4)
N4—H9N···Cl7	1.04 (6)	2.70 (5)	3.199 (3)	110 (3)
N4—H9N···Cl8	1.04 (6)	2.52 (6)	3.541 (3)	166 (4)
N4—H10N···Cl11^iii^	0.87 (5)	2.45 (4)	3.240 (4)	152 (4)
C4—H4···Cl11	0.93	2.83	3.621 (3)	144
C7—H7B···Cl1^v^	0.97	2.80	3.711 (3)	156
C22—H22···Cl2^vi^	0.93	2.69	3.602 (4)	166
C26—H26B···Cl8	0.97	2.82	3.589 (3)	137
C27—H27B···Cl5	0.97	2.81	3.610 (3)	140

Symmetry codes: (i) *x*−1, *y*+1, *z*−1; (ii) −*x*+1, −*y*+1, −*z*; (iii) −*x*+1, −*y*+1, −*z*+1; (iv) *x*−1, *y*, *z*; (v) *x*, *y*+1, *z*−1; (vi) −*x*+1, −*y*, −*z*+1.
